# CAR-T Access Disparities for Multiple Myeloma in the Midwest: A Social Determinants of Health Perspective

**DOI:** 10.3390/curroncol32090495

**Published:** 2025-09-03

**Authors:** Michael Weise, Shebli Atrash, Briha Ansari, Muhammad Umair Mushtaq, Joseph McGuirk, Al-Ola Abdallah, Zahra Mahmoudjafari, Nausheen Ahmed

**Affiliations:** 1 Division of Hematologic Malignancies & Cellular Therapeutics, University of Kansas Medical Center, Westwood, KS 66205, USA; m69479@kumc.edu (M.W.); mmushtaq@kumc.edu (M.U.M.); jmcguirk@kumc.edu (J.M.); zmahmoudjafari@kumc.edu (Z.M.); nahmed5@kumc.edu (N.A.); 2 Levine Cancer Institute, Atrium Health Wake Forest University School of Medicine, Charlotte, NC 28204, USA; shebli.atrash@atriumhealth.org; 3 Department of Biostatistics, Johns Hopkins University Bloomberg School of Public Health, Baltimore, MD 21205, USA; bansari1@jhu.edu

**Keywords:** multiple myeloma, CAR-T therapy, social determinants of health

## Abstract

Background: Multiple Myeloma (MM) is the most common type of blood cancer among black individuals. CAR-T therapy is crucial, but often inaccessible to many black patients and those from underserved communities. The University of Kansas Health System administers over 100 CAR-T treatments annually and aims to evaluate barriers to CAR-T therapy access related to the social determinants of health in the Midwest area. Methods: This study examined patients with MM referred for CAR-T therapy from January 2021 to December 2023, assessing how race, socioeconomic status, and insurance influenced eligibility for leukapheresis. Data on income and travel were gathered from the 2022 US Census and analyzed using R software. Results: The study included 271 referrals for MM CAR-T therapy involving 179 patients, with a median age of 66 years (51% male). Demographics: 80% white, 16% black, 2.2% other races, 1.8% Asian, with a median income of $70,644. Nearly half lived more than 30 min from the center (Mainly from Kansas, Missouri and Nebraska). Apheresis rates were similar across racial groups: 54% for whites, 54% for blacks, and 50% for others, while none of the three Asian patients proceeded. Nine patients (5%) could not proceed because of caregiver or insurance barriers, and cell collection rates were comparable regardless of distance (34% vs. 35%). Conclusion: This study showed that black representation in CAR-T access matches local demographics, indicating less disparity among minorities. Unlike national reports, distance, income, and insurance do not significantly affect access, suggesting the need for a national study on the social determinants impacting CAR-T access for multiple myeloma.

## Simple Summary

CAR-T therapy is a promising treatment for multiple myeloma, but access can be difficult, especially for people from underserved backgrounds. This study looked at patients referred for CAR-T therapy at a large Midwest medical center to understand whether factors like race, income, insurance, or distance from the hospital affected access. Surprisingly, access rates were similar for black and white patients, and distance or lower income did not seem to limit treatment. While some patients could not proceed due to caregiver or insurance issues, these cases were few. These findings suggest that strong support systems at the institutional level—such as help with housing and navigating insurance—can reduce barriers. This contrasts with national trends showing wider gaps and highlights the potential for policies that support patient navigation and local housing options. Future studies should explore access for patients who are never referred at all and look at how to bring this model to other regions.

## 1. Introduction

Chimeric antigen receptor T-cell (CAR-T) therapy represents a transformative advancement in the treatment of relapsed and refractory multiple myeloma (RRMM). CAR-T therapy involves the collection and genetic modification of patient’s cells to recognize and eliminate malignant plasma cells expressing B-cell maturation antigen (BCMA). In contrast to conventional chemotherapy and continuous immunomodulatory regimens, CAR-T offers the potential for deep and durable responses through a single infusion. Two BCMA-directed CAR-T therapies, idecabtagene vicleucel (ide-cel) and ciltacabtagene autoleucel (cilta-cel), are FDA-approved in the United States, demonstrating response rates of 73% and 97%, respectively [1,2]. These compelling results have positioned CAR-T cells as an essential treatment option for patients with advanced multiple myeloma.

Despite its promise, CAR-T cell therapy is not universally accessible. Barriers to access include the complex infrastructure requirements of therapy, the limited availability of authorized treatment centers, stringent clinical and logistical eligibility criteria, and significant financial burdens. These barriers are particularly pronounced among patients affected by adverse social determinants of health (SDOH), such as low socioeconomic status, limited health literacy, geographic isolation, underinsurance, or lack of insurance, and racial or ethnic minority status [3,4].

Administering CAR-T therapy requires a highly specialized and resource-intensive setting. Patients must undergo leukapheresis, lymphodepletion, and close monitoring post-infusion because of potentially life-threatening toxicities such as cytokine release syndrome (CRS) and immune effector cell-associated neurotoxicity syndrome (ICANS). These steps require access to multidisciplinary care teams, financial navigators, and social support resources. Furthermore, many centers, particularly those in rural or underserved regions, lack the necessary infrastructure to offer CAR-T therapy, which requires patients to travel long distances, often at significant financial and personal costs [5,6].

Socioeconomic disparities in cancer care have long been established, and multiple myeloma is no exception. African Americans are twice as likely to develop MM compared to white individuals (15.8 vs. 6.9 per 100,000), yet studies suggest that they are less likely to receive novel therapies, including CAR-T [3,7]. Additionally, patients with Medicaid or no insurance may encounter administrative and financial roadblocks, such as delayed approvals or outright denial of coverage, even if they are clinically eligible [4,8]. Factors such as caregiver availability, housing requirements, and proximity to treatment centers further contribute to access inequities [9,10].

The University of Kansas Health System (TUKHS), an academic center and regional CAR-T hub, is uniquely positioned to investigate CAR-T access in the context of SDOH. This study aimed to evaluate whether race, insurance status, and socioeconomic proxies such as household income and geographic proximity influenced the likelihood of patients with MM proceeding to leukapheresis. In doing so, we hope to inform future efforts aimed at expanding equitable access to CAR-T cell therapy in both urban and rural populations.

## 2. Methods

This retrospective, single-center study included all patients referred to the CAR-T therapy program at the TUKHS between January 2021 and December 2023. Data were extracted from institutional referral logs and electronic health records. Each patient was counted once; multiple referrals were recorded but not analyzed separately unless unique in nature.

Our center is one of the few institutions in the Midwest region that provides CAR-T cell therapy. We have established a centralized referral pathway in which most patients with multiple myeloma are referred from community oncology clinics for evaluation by our team at TUKHS, regardless of their initial eligibility status. This approach ensures that all referred patients are assessed directly by TUKHS oncologists, enabling optimal patient care, timely identification of appropriate candidates, and minimizing potential disparities in access to CAR-T therapy.

The primary outcome was the completion of leukapheresis or administration of an investigational allogeneic CAR-T product. The predictor variables included race, insurance type, geographic distance from the center, and median household income based on the ZIP code using data from the U.S. Census Bureau.

Geographic access was categorized by drive time to the institution as ≤30 min, 31–120 min, and >2 h (mainly from Kansas, Missouri, and Nebraska). Income was classified relative to the median cohort ($70,364). Insurance was categorized as Medicaid, Medicare, Commercial, or Other. Racial categories were recorded as white, African American (AA), Asian, or other.

TUKHS requires patients receiving CAR-T cells to have 24/7 caregiver support for the first 30 days post-infusion and to remain within 30 min of the center. Patients living farther must arrange temporary housing, often with institutional assistance through programs such as the American Cancer Society’s Hope Lodge program.

Descriptive statistics were used to summarize the demographic and clinical characteristics. Outcomes were analyzed using frequency distributions and stratified using SDOH indicators. Statistical analyses were conducted using R (R Core Team, 2024. R: A Language and Environment for Statistical Computing. R Foundation for Statistical Computing, Vienna, Austria. Available online: https://www.R-project.org/, accessed on 1 June 2024). Continuous variables were summarized using mean ± standard deviation (SD) for normally distributed data, or median and interquartile range (IQR) for non-normally distributed data. Differences between groups were assessed using analysis of variance (ANOVA) for comparisons involving more than two groups. Student’s *t*-test was applied for normally distributed variables, while the Wilcoxon-Mann-Whitney test was used for non-parametric comparisons. Categorical variables were presented as counts and percentages and compared using the Chi-squared test or Fisher’s exact test, as appropriate. Generalized linear regression was employed to assess associations between successful CAR-T cell administration and relevant clinical or treatment-related variables.

## 3. Results

Between 2021 and 2023, 179 patients with multiple myeloma were referred for CAR-T therapy at our institution, from a pool of 271 total referrals. The median age of the patients was 66 years (range, 34–85 years), and 33% had more than one referral. The cohort was predominantly white (80%), with African Americans accounting for 16%, and the remaining 4% comprised Asian and other races. The median estimated household income by ZIP code was $70,364 (range $31,250–$207,534), and 50.2% of patients resided within a 30 min driving distance from the institution. The majority of patients were insured through Medicare (71.5%), with the remainder covered by commercial insurance or Medicaid (See Table 1).

At the time of referral, 67% of the patients had a high risk. The median number of prior treatment lines was five (1–12). Approximately 83% of the patients were triple-class refractory and 14% were penta-refractory.

**Figure 1:**
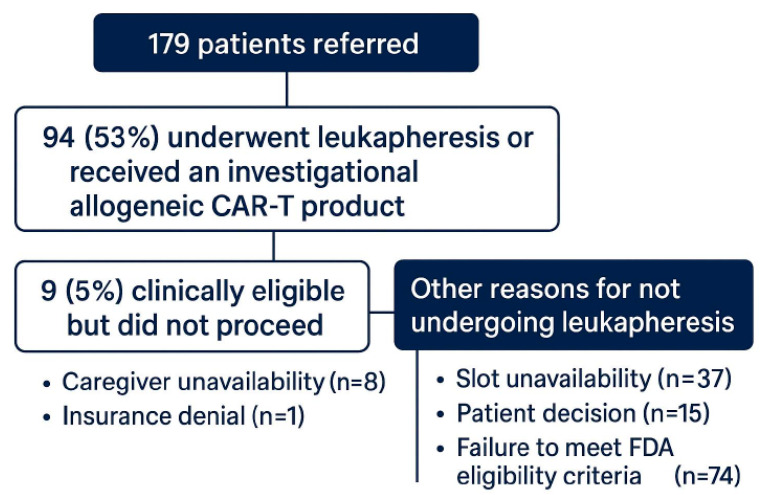
Flow diagram of CAR-T therapy referral outcomes.

Of the 179 patients referred, 94 (53%) underwent leukapheresis or received an investigational allogeneic CAR-T product. Among these, 50 (53%) were treated with idecabtagene vicleucel, 39 (41%) with ciltacabtagene autoleucel, and 5 (6%) with an allogeneic CAR-T therapy. Nine patients (5%) were clinically eligible but did not proceed due to caregiver unavailability (n = 8) or insurance denial (n = 1). Other reasons for not undergoing leukapheresis included slot unavailability (n = 37), patient decision (n = 15), and failure to meet the FDA eligibility criteria (n = 74) (See Figure 1).

### 3.1. Geographic Access

**Figure 2:**
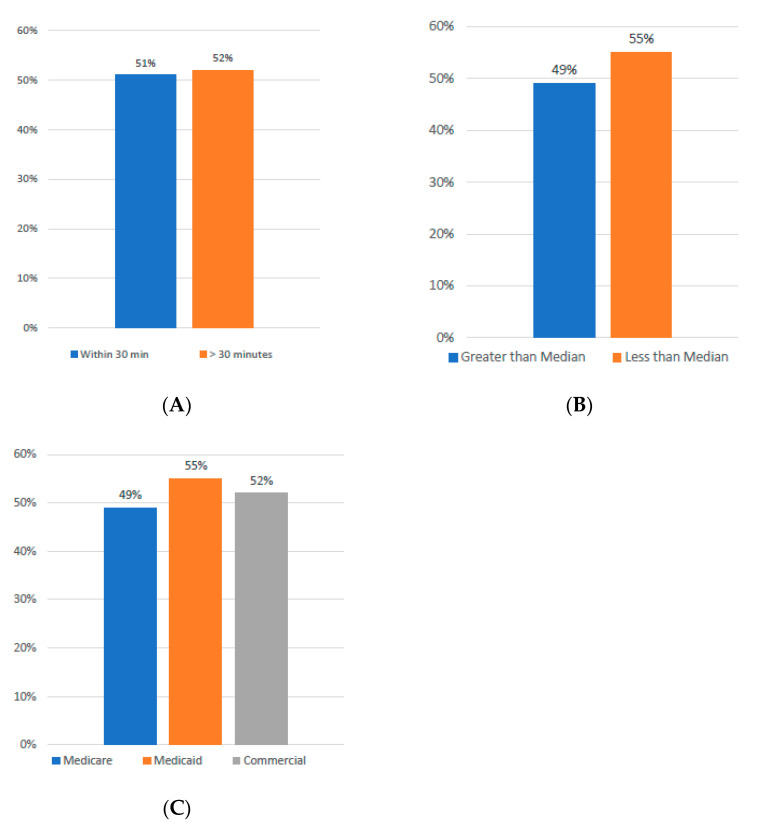
(**A**) Incidence of Leukapheresis by Driving Distance. (**B**) Incidence of Leukapheresis Relative to Income. (**C**) Incidence of Leukapheresis by Insurance Type.

Nearly half of the patients referred for treatment, accounting for 50%, resided within a convenient 30 min radius of the treatment center. Interestingly, the leukapheresis rate was quite comparable, standing at 51% for those living closer and slightly higher at 52% for those who traveled from greater distances. This data suggest that geographic distance was not a significant barrier to treatment in this cohort (See Figure 2A).

### 3.2. Income and Insurance

When analyzed by ZIP-code-level income, patients from areas with incomes below the median household income underwent leukapheresis at a rate of 55%. In contrast, patients from higher-income ZIP codes had a leukapheresis rate of 49%. Additionally, when examining the data by insurance type, Medicaid-insured patients had the highest leukapheresis rate at 55%, followed by those with commercial insurance at 52% and Medicare beneficiaries at 49%. These findings indicate that income and insurance type did not negatively affect access to treatment (Figure 2B,C).

### 3.3. Racial Distribution

**Figure 3:**
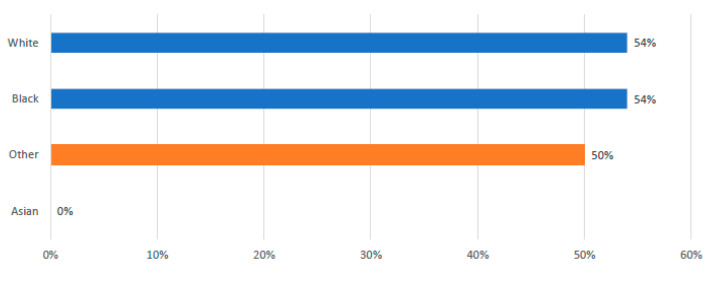
Incidence of Leukapheresis by Race.

The rates of leukapheresis were found to be comparable between White and African American patients, with both groups showing a rate of 54%. In contrast, no Asian patients, numbered just three, underwent the leukapheresis procedure; however, due to the limited sample size, it remains challenging to draw any definitive conclusions regarding this demographic (See Figure 3).

### 3.4. Univariate Predictors of CAR-T Infusion

In univariate analyses (Table 2), no sociodemographic or access factor predicted receipt of infused CAR-T—distance/driving time from Westwood, ZIP code income, relationship status, insurance type, race, employment, caregiver availability, patient choice, or age (all *p* ≥ 0.14). The only significant predictor was the number of prior therapy lines (OR 1.30 per line; 95% CI 1.09–1.47; *p* = 0.003).

## 4. Discussion

This single-institution analysis found no significant disparities in access to CAR-T therapy based on race, income bracket, or insurance status among the referred patients. While national data have reported substantial inequities in CAR-T access, particularly among racial and ethnic minorities and low-income groups, our findings suggest that institutional factors, such as patient navigation, housing support, and coordinated referral pathways, may help mitigate these disparities [3,4,9]. TUKHS’ role as a major CAR-T referral center likely contributed to more uniform referral practices and consistent patient follow-up.

Similar rates of leukapheresis across racial groups are particularly notable given the higher MM incidence among African Americans and the documented underutilization of novel therapies in this population [7]. Previous studies have shown that African American patients are less likely to be referred for autologous stem cell transplantation or clinical trials, often because of systemic biases, access issues, or healthcare mistrust [3,9]. Our study found no racial-based difference in CAR-T access, which may indicate progress in institutional equity initiatives; however, the sample size may limit broader conclusions.

Leukapheresis rates were not adversely affected by insurance status. Medicaid patients, often considered more vulnerable due to their lower income and higher rates of comorbidity, had the highest rate of CAR-T access (55%). This could reflect institutional efforts to work proactively with insurers and provide social support services that address nonclinical needs. However, 5% of otherwise eligible patients were unable to proceed due to insurance denials or a lack of caregiver support, highlighting that such barriers persist even in resource-rich environments [8,10].

Interestingly, geographical proximity did not significantly influence access. This may be attributable to temporary lodging support through initiatives such as the Hope Lodge or institutional housing funding. However, geographic distance remains a well-established barrier at the national level, particularly in rural regions without nearby CAR-T centers [5,6,11].

One critical limitation of our study was its inability to account for patients who were never referred for CAR-T consultation. Many patients from underserved communities may never reach a tertiary care center because of provider bias, limited awareness, or logistical limitations [6,11]. This “invisible denominator” is vital to a complete understanding of access inequities and must be addressed in future multicenter prospective studies. Additionally, using the ZIP code income as a proxy for individual SES may misclassify patients, especially in socioeconomically diverse metropolitan areas.

Structural and systemic barriers continue to affect access. The institutional requirements for caregiver availability and local housing may inadvertently exclude eligible patients from receiving care. Furthermore, patients’ decisions not to pursue CAR-T may reflect deeper issues of trust, cultural perception, or a limited understanding of the therapy’s benefits and risks, highlighting the importance of shared decision-making and culturally sensitive education.

Recent policy changes may improve access. In June 2025, the FDA eliminated the Risk Evaluation and Mitigation Strategy (REMS) requirements for all approved autologous CAR-T therapies, including ide-cel and cilta-cel [12]. This change reduced post-infusion monitoring burdens, including the requirement for patients to remain near a certified treatment center and restrictions on driving after infusion. Such regulatory flexibility could ease the patient burden, particularly for those in rural areas, and allow more community-based CAR-T expansion.

Going forward, broader solutions must include expanding CAR-T center networks, reducing payer variability, increasing provider education, and improving early referrals from community practices. Patient navigation programs, financial counseling, and partnerships with social service agencies are vital for supporting vulnerable populations.

We plan to collaborate with additional Midwest institutions to launch a prospective multicenter study. This initiative will help identify regional variability in access and build a consensus on best practices to ensure equitable CAR-T delivery across diverse patient populations.

## 5. Conclusions

CAR-T therapy represents a paradigm shift in the treatment of RRMM, but its success depends not only on clinical efficacy but also on equitable access. Our institutional experience suggests that thoughtful infrastructure, support services, and coordinated care pathways can help reduce the disparities associated with SDOH. However, broader systemic reforms and prospective data are needed to ensure that all patients, regardless of race, income, or location, can benefit from life-saving therapy.

## Figures and Tables

**Table 1:** Baseline Characteristics of patients with RRMM.

Patients Characteristics	All Patients (n = 179)	*p*-Value
Age, years, median (range)	66 years (34–85)	0.6
Gender, male:female	91:88	0.09
Race, no of patients (%)	0.031	
Caucasian	144 (80%)	
Black	28 (16%)	
Other	4 (2.2%)	
Asian	3 (1.7%)	
MM paraprotein, no. of patients (%)	>0.9	
IgG	111 (62%)	
Non-IgG	40 (22%)	
Light chain	28 (16%)	
Median Previous Lines of Treatment (range)	5 (1–12)	0.1
* High Risk Cytogenetics	120 (67%)	0.7
Zip Code Income (Median)	$70,364 (31,250–207,534) 0.07	
Resides within 30 min of TUKHS	90 (50.2%)	<0.001
Insurance		
Commercial	51 (28.6%)	
Medicare	117 (65.3%)	
Medicaid	11 (6.1%)	

* High Risk Cytogenetics: including patients with either: deletion P17 or TP53 mutation, t(4;14), t(14;16), t(14;20) and 1q abnormality.

**Table 2:** Univariate analysis for infused CAR-T treatment.

Characteristic	N	OR	95% CI	*p*-Value
Driving time from Westwood	179	1	0.99, 1.00	0.2
Median Income for Zip in 2022	179	1	1.00, 1.00	0.9
Relationship Status				
Divorced	12	—	—	
Married	126	0.7	0.26, 1.67	0.4
Single	32	0.4	0.14, 1.32	0.14
Widowed	9	0.8	0.16, 3.37	0.7
Medicare	122	0.8	0.48, 1.39	0.4
Medicaid	11	1.1	0.38, 3.12	0.8
Commercial	48	1	0.58, 1.79	>0.9
Race				
Other	34	—	—	
White	145	1.1	0.58, 2.16	0.8
Employed at time of consult				
No	133	—	—	
Yes	46	0.9	0.48, 1.48	0.6
Lack of caregiver	6	0		>0.9
Patient choice	11	0		>0.9
distance	179	1	0.99, 1.00	0.2
Driving				
30 min	90	—	—	
less than 30 min	89	1	0.62, 1.71	>0.9
Age at Consult	179	1	0.97, 1.02	0.6
Previous lines of therapy prior to consult	179	1.3	1.09, 1.47	0.003
Abbreviations: CI = Confidence Interval, OR = Odds Ratio				

## Data Availability

The data presented in this study are available on request from the corresponding author.
